# Elucidating the role of ferrous ion cocatalyst in enhancing dilute acid pretreatment of lignocellulosic biomass

**DOI:** 10.1186/1754-6834-4-48

**Published:** 2011-11-10

**Authors:** Hui Wei, Bryon S Donohoe, Todd B Vinzant, Peter N Ciesielski, Wei Wang, Lynn M Gedvilas, Yining Zeng, David K Johnson, Shi-You Ding, Michael E Himmel, Melvin P Tucker

**Affiliations:** 1 Biosciences Center, National Renewable Energy Laboratory, Golden, CO 80401, USA; 2 National Bioenergy Center, National Renewable Energy Laboratory, Golden, CO 80401, USA

**Keywords:** dilute acid pretreatment, iron cocatalyst, ferrous ions, metal cocatalyst, biomass, cellulose, corn stover, cotton linter, filter paper, Fourier transform, Raman spectroscopy

## Abstract

**Background:**

Recently developed iron cocatalyst enhancement of dilute acid pretreatment of biomass is a promising approach for enhancing sugar release from recalcitrant lignocellulosic biomass. However, very little is known about the underlying mechanisms of this enhancement. In the current study, our aim was to identify several essential factors that contribute to ferrous ion-enhanced efficiency during dilute acid pretreatment of biomass and to initiate the investigation of the mechanisms that result in this enhancement.

**Results:**

During dilute acid and ferrous ion cocatalyst pretreatments, we observed concomitant increases in solubilized sugars in the hydrolysate and reducing sugars in the (insoluble) biomass residues. We also observed enhancements in sugar release during subsequent enzymatic saccharification of iron cocatalyst-pretreated biomass. Fourier transform Raman spectroscopy showed that major peaks representing the C-O-C and C-H bonds in cellulose are significantly attenuated by iron cocatalyst pretreatment. Imaging using Prussian blue staining indicated that Fe^2+ ^ions associate with both cellulose/xylan and lignin in untreated as well as dilute acid/Fe^2+ ^ion-pretreated corn stover samples. Analyses by scanning electron microscopy and transmission electron microscopy revealed structural details of biomass after dilute acid/Fe^2+ ^ion pretreatment, in which delamination and fibrillation of the cell wall were observed.

**Conclusions:**

By using this multimodal approach, we have revealed that (1) acid-ferrous ion-assisted pretreatment increases solubilization and enzymatic digestion of both cellulose and xylan to monomers and (2) this pretreatment likely targets multiple chemistries in plant cell wall polymer networks, including those represented by the C-O-C and C-H bonds in cellulose.

## Background

Enzymatic biomass conversion enabled by dilute acid pretreatment processes has been studied for many years but remains one of the key obstacles to the economical production of lignocellulosic biofuels today. Ferrous ion (hereinafter referred as Fe^2+^) enhancement of dilute acid pretreatment of biomass is a promising technology that enhances the release or conversion of sugars during pretreatment [1]. The economic benefit of adding Fe^2+ ^ions can be realized by reducing the severity of pretreatment conditions (a composite factor based on acid concentration, temperature and time) while retaining comparable conversion to biomass sugars.

Fourier transform infrared spectroscopy (FTIR) was used to study the interaction between first-row transition metal ions (to which Fe belongs) and glucose in the glassy state. The results showed that all bands of sugar skeletal vibration modes and all C-O-H and C-O vibration modes of glucose were shifted in metal ion/D-glucose complexes [2]. In another study, FTIR spectra also showed that the metal ions (including Fe^2+ ^ions) induced changes in the C-C and C-O ring as well as in the skeletal modes of xylose [3].

Researchers in recent studies have reported that the ferric form of iron (FeCl_3_) also is efficient in functioning as a catalyst for releasing hemicellulose from corn stover (CS) by hot water pretreatment [4] and that the enzymatic digestibility and cellulose recovery can be enhanced after hot ethanosolv/FeCl_3 _pretreatment of barley straw [5]. Most recently, it was reported that Fe^2+ ^and Fe^3+ ^ions are effective in enhancing the Lewis acid cocatalyzed dilute sulfuric acid pretreatment of lignocellulosic biomass [6].

However, the precise mechanism underpinning the facilitation of biomass deconstruction as catalyzed by iron ions, either in Fe^2+ ^ion form [1,6] or in ferric ion form (hereinafter referred to as Fe^3+^) [4-6], remains unknown. Adding to the technical difficulty of studying the role of Fe^2+ ^ions in biomass pretreatment is its transient nature. Fe^2+ ^ions can easily be oxidized to Fe^3+ ^ions by exposure to air. Thus continuous argon or nitrogen gas needs to be used to purge the Fe^2+ ^ion-containing solutions until it is transferred to a sealed container, such as reactors used in pretreatment. Consequently, there is a scarcity of information about which components of biomass, a complex matrix of celluloses, hemicelluloses and lignins, are affected by iron cocatalysts and which types of chemical bonds are actively engaged during catalytic deconstruction.

The aim of our research was to identify the factors that may contribute to metal-enhanced efficiency during dilute acid/Fe^2+ ^ion pretreatment and initiate the exploration of their mechanisms by using model cellulose substrates (filter paper (FP) and cotton linter (CL)) as well as model biomass feedstock (corn stover). To achieve this goal, we employed high-performance liquid chromatography (HPLC), dinitrosalicylic acid (DNS) assay, Fourier transform (FT) Raman spectroscopy, Prussian blue iron staining, laser dissection microscopy, transmission electron microscopy (TEM) and scanning electron microscopy (SEM) to study dilute acid/Fe^2+ ^ion pretreatment and its effect on the digestibility of biomass residues.

## Results

### Biophysical roles of Fe^2+ ^ions in cellulose pretreatment: precipitation patterns of pretreated filter paper

**Figure 1:**
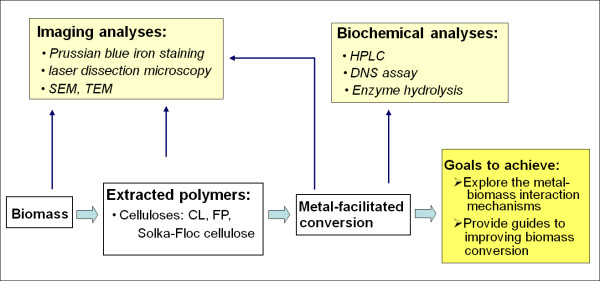
**Diagram showing the experimental plan**. Imaging and analytical tools were used to examine the metal ion-biomass interaction at different stages of biomass processing and conversion. Biochemical analytical methods were used to measure the efficacy of metal ions in enhancing biomass conversion. CL = cotton linter; DNS = dinitrosalicylic acid; FP = filter paper; HPLC = high-performance liquid chromatography; SEM = scanning electron microscope; TEM = transmission electron microscopy.

The overall experimental approach is illustrated in Figure 1. The first step toward understanding the role of the Fe^2+ ^ion cocatalyst in enhancing biomass degradation was to investigate the effects of dilute acid/Fe^2+ ^ion pretreatments on the model celluloses, that is, FP.

**Figure 2:**
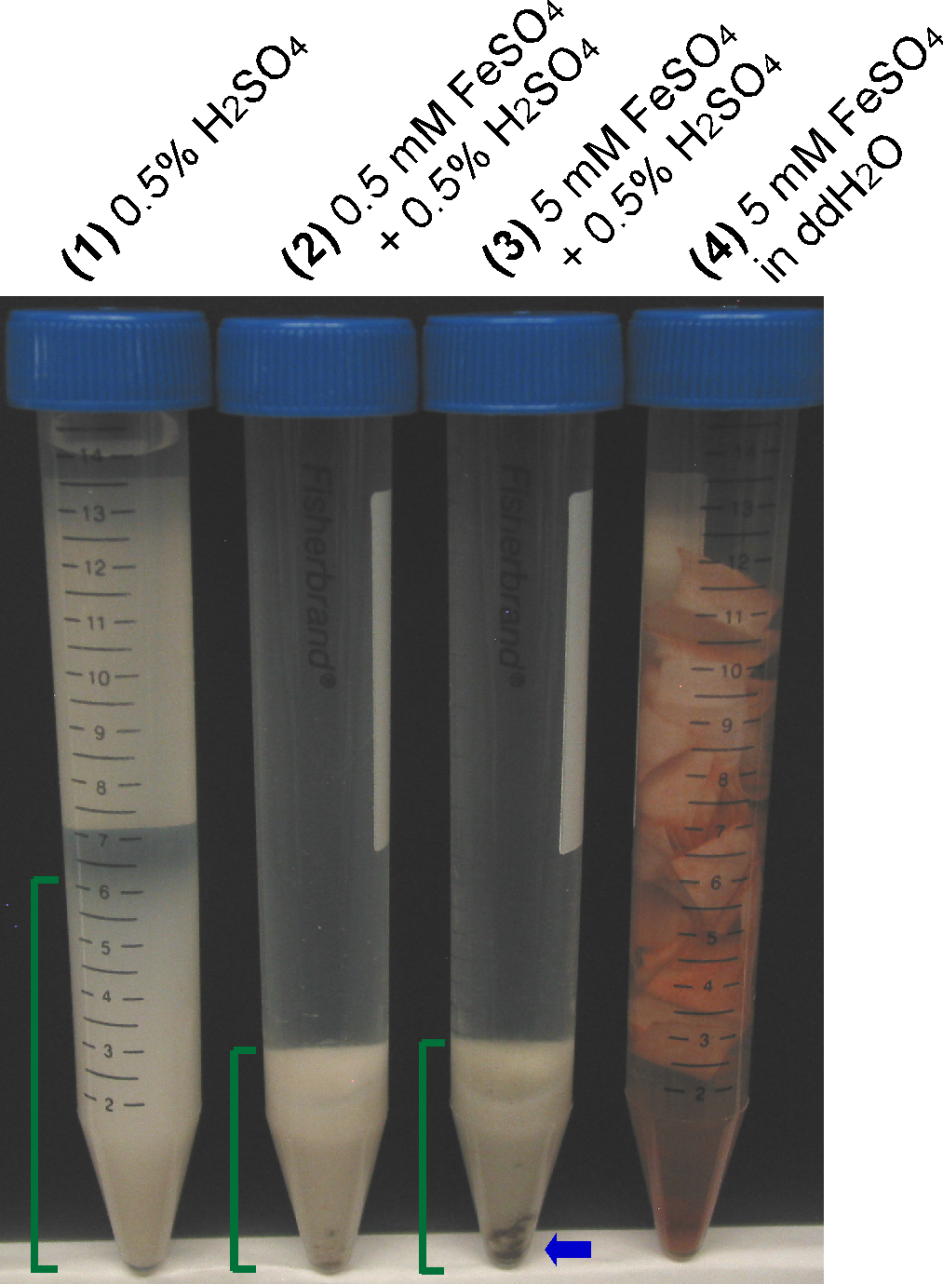
**Filter paper strips pretreated in pipe reactors at 190°C for five minutes in the presence of 0.5% H_2_SO_4_, 0.5 to 5 mM FeSO_4 _or a combination of acidic and salt chemical solutions**. All samples were pretreated at the same time.

After pretreatment of FP in gold-plated pipe reactors at 190°C for five minutes in the presence of 0.5 wt% H_2_SO_4_, with or without Fe^2+^, the remaining FP was pelleted by centrifugation, resuspended in distilled deionized water (ddH_2_O) and allowed to stand overnight. The dilute acid-alone pretreatment caused disintegration of the FP strips by disrupting the strips into fiber powders (Figure 2, tube 1). Similarly, pretreatment with the addition of 0.5 mM and 5 mM FeSO_4 _into 0.5 wt% H_2_SO_4 _(Figure 2, tubes 2 and 3, respectively) also caused the strips to disintegrate into fiber powders. Differently from the dilute acid control, a black precipitate formed in the pretreatment reaction after the 5 mM FeSO_4 _addition, which suggests that a portion of the Fe ions may have been precipitating out of the solution as black ferrous oxide (FeO) or ferrous-ferric oxide (Fe_3_O_4_) in the presence of dilute acid (see the bottom of tube 3 in Figure 2, as indicated by the blue arrow). Previous reports have described the color of ferrous oxide or ferrous-ferric oxide being black [7-9]. In contrast, the color of ferric oxide (Fe_2_O_3_) is reddish brown [9]. Further investigation is needed to determine whether this black precipitate is also formed in "absolute" control (that is, 5 mM FeSO_4 _in 0.5 wt% H_2_SO_4 _but no FP) under the same pretreatment conditions, as well as the exact chemical nature of this precipitate.

Meanwhile, the FP strips in tube 4 were pretreated with 5 mM Fe^2+ ^in ddH_2_O, and the formation of red ferric oxide (Fe_2_O_3_, that is, rust) observed throughout the FP strips, especially at the edges of the strips, is likely due to the oxidation of Fe^2+ ^to Fe^3+ ^ions (Figure 2, tube 4).

The difference in precipitation volume and appearance of cellulose residues may indicate that FeSO_4 _physicochemically modifies the remaining FP in a way that leads to changes in the surface charge and/or hydrophobicity of FP during dilute acid/metal pretreatment.

### Sugar released during pretreatment of filter paper and cotton linter

We assessed three aspects of FP and CL pretreatment efficiency: (1) how much sugar was released, (2) the chemical and physicochemical changes in the remaining FP and CL and (3) the enzymatic digestibility of the remaining FP and CL. HPLC was used to measure the released sugars in the hydrolysate liquors of pretreated samples.

**Figure 3:**
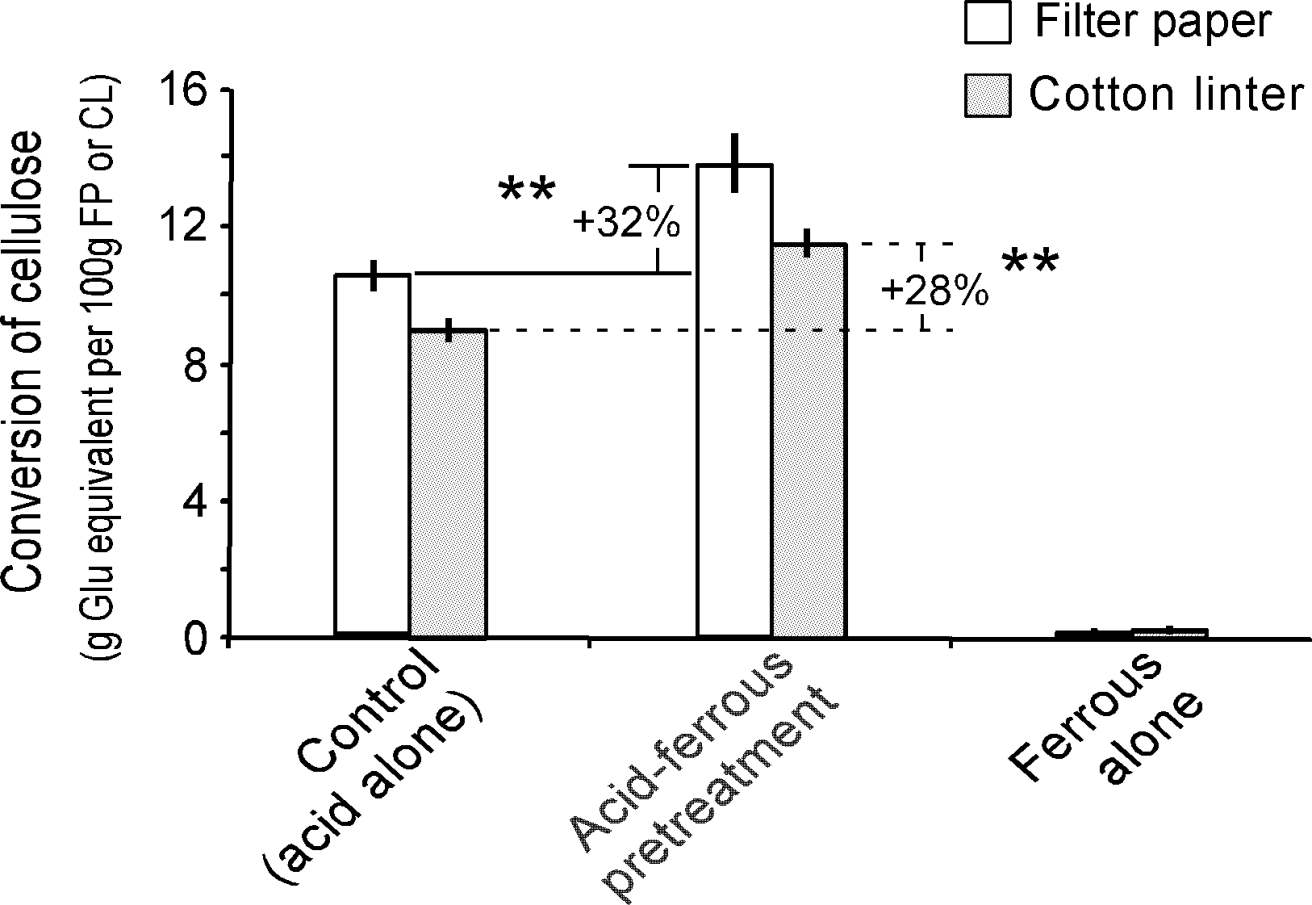
**The release of sugars from filter paper and cotton linter by dilute acid and/or ferrous ion pretreatments at 190°C for five minutes**. The released sugars, including both glucose and cellobiose (together glucose equivalent) and expressed in percentage of conversion of filter paper (FP) and cotton linter (CL) celluloses, were quantitated by HPLC analysis of the supernatants from the pretreatment of filter paper strips and cotton linter disks. Values are presented as the mean (± SE) of three replicates for each of the pretreatments. **P < 0.01 indicates statistical significance. Glu = glucose; HPLC = high-performance liquid chromatography.

Figure 3 shows the release of glucose and cellobiose as a result of the pretreatment of FP strips or CL disks with or without Fe^2+ ^or acid addition. Compared with dilute acid-alone pretreatment, the dilute acid/Fe^2+ ^ion pretreatment was more efficient in releasing sugars from FP and CL, showing enhancement of 28% to 32%, for glucose and cellobiose released during pretreatment. In contrast, pretreatment of FP strips and CL disks in ddH_2_O with 5 mM Fe^2+ ^(no acid) resulted in less than 10% of the glucose and cellobiose release found with the dilute acid control alone.

### Quantification of reducing sugar ends in remaining filter paper and cotton linter

A DNS assay was carried out to measure the chemical changes (specifically, the amount of reducing sugar ends) in the remaining FP and CL. Table 1 reports the results of DNS reducing sugar assays performed on 0.2-g (dry weight basis) washed samples of pretreated FP and CL disk residues following pretreatment with or without added Fe^2+ ^ions. The estimated number of reducing ends measured by the DNS assay within the FP and CL fiber residues pretreated in the presence of Fe^2+ ^increased 20% to 32% over the dilute acid control. For pretreatment with Fe^2+ ^in ddH_2_O alone, the generation of reducing ends was much less than that for the dilute acid control. The results shown in Table 1 indicate that the addition of Fe^2+ ^ions to dilute acid pretreatments caused additional scissions of the β(1→4)-glycosidic bond within the cellulosic fibers for both FP and CL.

**Table 1:** The generation of reducing sugar ends from filter paper and cotton linter after dilute acid and/or metal pretreatments

Pretreatments	Reducing sugar ends^a^	Average increase or decrease over control
FP control (0.5% H_2_SO_4_)	1.92 ± 0.07	
FP (5 mM FeSO_4 _in ddH_2_O)	0.88 ± 0.05	-54%**
FP (0.5% H_2_SO_4 _+ 0.5 mM FeSO_4_)	2.47 ± 0.13	28%**
FP (0.5% H_2_SO_4 _+ 5 mM FeSO_4_)	2.32 ± 0.16	20%*
CL control (0.5% H_2_SO_4_)	1.68 ± 0.06	
CL (5 mM FeSO_4 _in ddH_2_O)	1.04 ± 0.05	-38%**
CL (0.5% H_2_SO_4 _+ 0.5 mM FeSO_4_)	2.23 ± 0.13	32%**
CL (0.5% H_2_SO_4 _+ 5 mM FeSO_4_)	2.17 ± 0.15	29%**

CL = cotton linter; ddH_2_O = distilled deionized water; FP = filter paper. ^a^Glucose equivalent in grams per 100 g of FP or CL residue. Values are presented as means ± SE of three replicate experiments for each of the pretreatments. *P < 0.05 and **P < 0.01 represent statistically significant difference from the control.

### Enzymatic digestibility of the remaining filter paper and cotton linter

**Figure 4:**
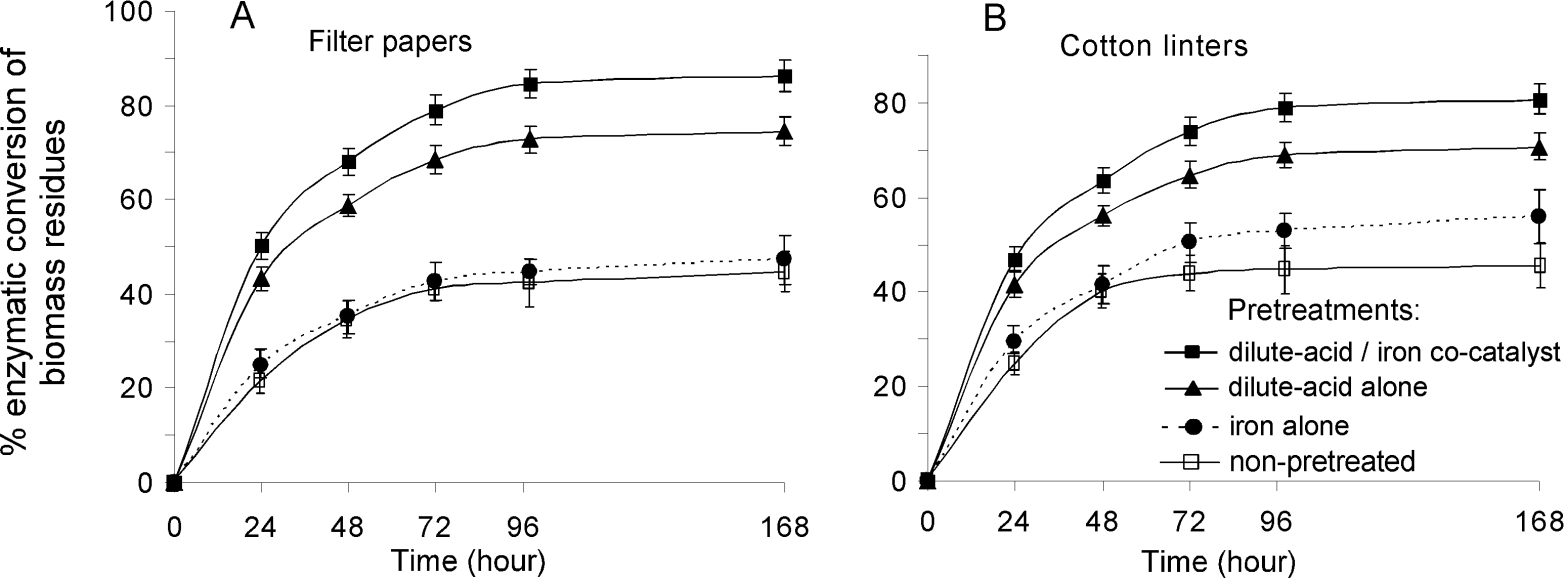
**Enzymatic digestibility of filter paper and cotton linter residues after different pretreatments**. **(A) **Glucose yield from filter paper (FP) and **(B) **glucose yield from cotton linter (CL) during 24 to 168 hours of enzymatic hydrolysis. The pretreatments for the biomass included dilute acid/iron cocatalyst (0.5 wt% H_2_SO_4 _+ 5 mM FeSO_4_), dilute acid alone (0.5 wt% H_2_SO_4_) and iron alone (5 mM FeSO_4_). For comparison purposes, the enzymatic hydrolysis of non-pre-treated FP and CL are also presented as "absolute" controls. Values are presented as means ± SE of glucose conversion (%) from three replicate experiments.

The glucose yield from enzymatic hydrolysis of different pretreated FP and CL materials is shown in Figure 4. The two dilute acid/iron cocatalyst-pretreated substrates, FP and CL, showed the highest glucose yield throughout the enzymatic hydrolysis process, reaching 87% ± 3% and 81% ± 3% of cellulose conversion, respectively, after 168 hours of digestion. In particular, the glucose yields from dilute acid/iron cocatalyst pretreatment increased by 16% for FP and by 14% for CL compared with the glucose yields from dilute acid-alone pretreatment. In contrast, there was no significant difference between 5 mM FeSO_4 _alone (in water)-pretreated and non-pre-treated FP and CL (Figures 4A and 4B, bottom two lines in each panel).

### Effect of Fe^2+ ^ions on cellulose degree of polymerization in acid pretreatment

It is known that dilute acid treatment can lead to a degree of polymerization (DP) reduction of cellulose [10]. To examine the effect on the DP of cellulose by adding metal ions to dilute acid pretreatment, a set of pretreatments (outlined in Table 2) was used to treat the Solka-Floc powdered cellulose (International Fiber Corp, North Tonawanda, NY, USA), followed by size exclusion chromatography (SEC) analysis. The results demonstrate that supplementation with Fe^2+ ^ions to the dilute acid can further reduce the DP of pretreated Solka-Floc cellulose (Table 2).

**Table 2:** Effect of iron catalyst on the degree of polymerization of Solka-Floc cellulose after various pretreatments

Pretreatments	Weighted average degree of polymerization
Untreated Solka-Floc cellulose (control)	1, 110
Dilute acid	260
Dilute acid/Fe^2+^	150

The cellulose samples were pretreated in either dilute acid (0.5 wt% H_2_SO_4_) or dilute acid/Fe^2+ ^(0.5 wt% H_2_SO_4_/5 mM FeSO_4_) at 160°C for five minutes. Each data point represents an average of two measurements.

Future studies are needed to confirm the DP measurement of the model cellulose materials, FP and CL. These DP data will be helpful for exploring whether there is any correlation between the DP and the measured reducing sugar ends in the pretreated FP and CL residues (as described in earlier section, "Quantification of reducing sugar ends in remaining filter paper and cotton linter".

### Fourier transform Raman spectra of pretreated model cellulose substrates

**Figure 5:**
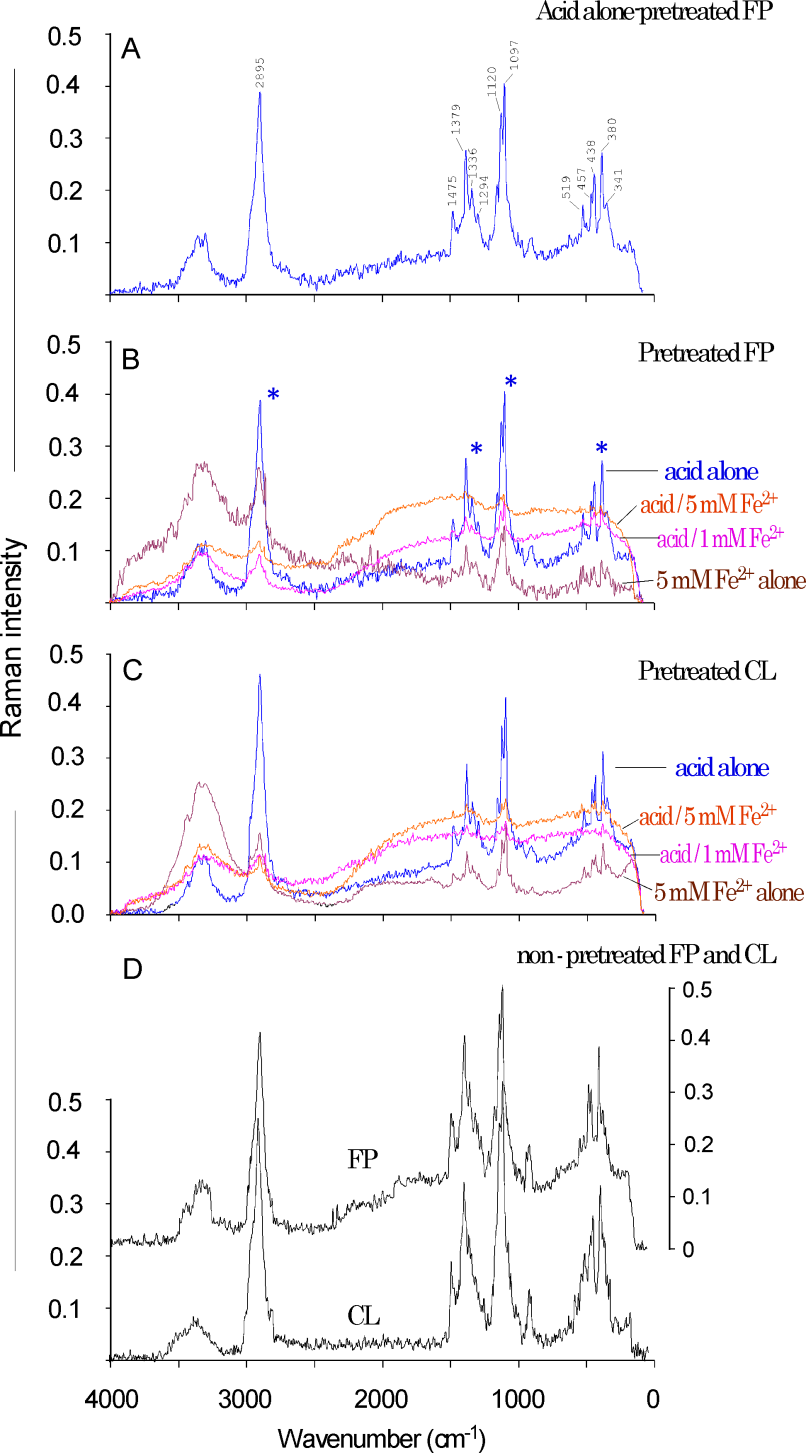
**The Fourier transform Raman spectra of filter paper and cotton linter celluloses after pretreatments with dilute acid and/or ferrous ions**. **(A) **Acid-alone (control) pretreated filter paper (FP) showing major peaks. **(B) **FP with various pretreatments. **(C) **Cotton linter (CL) with various pretreatments. **(D) **Non-pre-treated FP and CL. Asterisk in part **(B) **highlights the major peaks affected by the dilute acid/Fe^2+ ^ion pretreatment.

As a control, we measured the FT Raman spectra for the dilute acid alone-pretreated FP control (Figure 5A). Major peaks are labeled with their corresponding wave numbers for ease in matching the peaks with their assignments (Table 3). The FT Raman spectra of FP from four types of pretreatments across 4, 000/cm to 200/cm regions are shown in Figure 5B. Remarkably, several major peaks in dilute acid alone-pretreated FP (indicated by asterisks in Figure 5B, blue line) were attenuated or were not present in the spectra of dilute acid/1 or 5 mM Fe^2+ ^ion-pretreated FP (Figure 5B, red and pink lines). In contrast, these major peaks were still distinguishable in Fe^2+ ^ion alone-pretreated FP, despite the fact that their Raman intensities were lower (Figure 5B, brown line). These observations highlight the uniqueness of dilute acid/Fe^2+ ^ion pretreatment from pretreatment with either dilute acid alone or Fe^2+ ^ion alone.

**Table 3:** Frequency and characterization of major Fourier transform Raman peaks in spectra of dilute acid-alone pretreated filter paper and cotton linter samples were flattened in spectra of dilute acid/ferrous ion-pretreated samples

Pretreatment regions in four studies (peaks/cm)				
	
Current study	**Liu **[13]	**Edwards *et al *.**[12,28]	**Fechner *et al *.**[11]	Band assignment
Assigned peaks				
2, 895	n/a	n/a	2, 894	C-H stretching
1, 475	1, 476	1, 478		δ (CH2) scissors
1, 379	1, 380	1, 380		δ (CH2)
1, 336	1, 341	1, 338		δ (CH2) wagging, δ (C-O-H)
1, 294	1, 295	1, 294		δ (CH2) twisting
1, 120	1, 122	1, 122		γ (C-O-C) glycosidic bond, symmetric stretching
1, 097	1, 096	1, 097		γ (C-O-C) glycosidic bond, symmetric stretching
Unassigned peaks				
519, 457, 438, 380, 341				

n/a = not applicable.

Assignments of major FT Raman spectral peaks that were different between dilute acid alone-pretreated versus dilute acid/Fe^2+ ^ion-pretreated FP are given in Table 3. As illustrated in Figures 5A and 5B and as listed in Table 3 our FT Raman spectroscopy results suggest that the following bond types are likely to be the targets for the ferrous ions during the dilute acid/Fe^2+ ^ion pretreatment of FP and CL. First, the C-O-C glycoside link between the D-glucose units in cellulose covers the region of 1, 120/cm and 1, 097/cm, ascribed to the C-O-C glycosidic link symmetric and asymmetric stretching modes, respectively. These belong to backbone stretching vibrations. Second, the C-H glycoside bond covers the region of 1, 485/cm to 1, 270/cm as well as 2, 895/cm, corresponding to the C-H bending, stretching, twisting or wagging.

These observations are partially consistent with, and extend the knowledge gained from, a previous FTIR study in which bands of sugar skeletal C-O vibration modes of glucose were affected by metal ion/D-glucose complexes [2]. Furthermore, most bands in the spectra of the dilute acid/Fe^2+ ^ion-pretreated FP were broader than the bands in the spectra of dilute acid alone- or ferrous ion alone-pretreated bands, suggesting that the former contain a higher amount of amorphous regions [11]. Another indicator of the change in the cellulose crystalline structure comes from specific bands of 1, 160/cm, 1, 120/cm and 1, 097/cm, which have been used to trace changes in cellulose crystallinity [12,13]. Our results demonstrate that the bands of 1, 120/cm and 1, 097/cm were attenuated in dilute acid/Fe^2+ ^ion-pretreated samples compared to those from dilute acid alone pretreatment. Usually, the ranges of crystallinity for FP and CL are 40% to 50% and 65% to 80%, respectively [13]. We suggest that monitoring the possible effects of Fe^2+ ^ions on the crystallinity of cellulose during pretreatment should be considered in future studies.

It is also noteworthy that there is an underexplored range of 550/cm to 300/cm in the FT Raman spectra of cellulose samples. We observed a cluster of peaks in the range of 550/cm to 300/cm in dilute acid alone-pretreated FP (Figure 5B). This cluster of peaks, listed in Table 3 (bottom rows: 519/cm, 438/cm, 380/cm and 341/cm), is in agreement with similar previously published observations in microcrystalline and powdered cellulose [11]. Because these peaks were attenuated by dilute acid/Fe^2+ ^ion pretreatment, they are likely linked to the effects of the Fe^2+ ^ion cocatalyst on FP and CL during pretreatment. However, the scarcity of literature about the assignment of these bands to the structure of cellulose necessitates further investigation before well-supported conclusions may be drawn. Parallel studies were also conducted in which FT Raman spectra of CL after various pretreatments were used. The spectra in Figure 5C show that the CL was affected similarly to the FP (Figure 5B) under both control (acid alone) and other pretreatments.

We performed an additional FT Raman experiment on the non-pre-treated FP and CL. Their spectra, shown in Figure 5D, are similar to the dilute acid alone-pretreated FP and CL in Figures 5A through 5C, confirming the unique effect of dilute acid/Fe^2+ ^ion pretreatment in attenuating several major peaks in the FT Raman spectra of FP and CL. Furthermore, the FT Raman spectra of non-pre-treated FP and CL shown in Figure 5D are similar to the Raman spectra of Sigma cellulose powder (Sigma-Aldrich, St Louis, MO, USA) obtained from CL in a recent study [14], thereby partly validating our spectral data.

### Imaging plant cell wall components of native, non-pre-treated corn stover in the presence of Fe^2+ ^ions

In addition to investigating the biochemical and spectral effects of Fe^2+ ^ions on biomass conversion, we applied different imaging analysis approaches to investigate the direct interaction between metal ions and their biomass targets. We began these imaging studies by examining the binding and interaction of Fe^2+ ^ions with native, non-pre-treated CS. We hypothesized that Fe^2+ ^ions are likely to be bound with or adsorbed into one or multiple components of plant cell walls. As we carried out the Prussian blue staining assay, we set out to determine whether Fe^2+ ^ions are bound with or adsorbed into cellulose or lignin or both.

**Figure 6:**
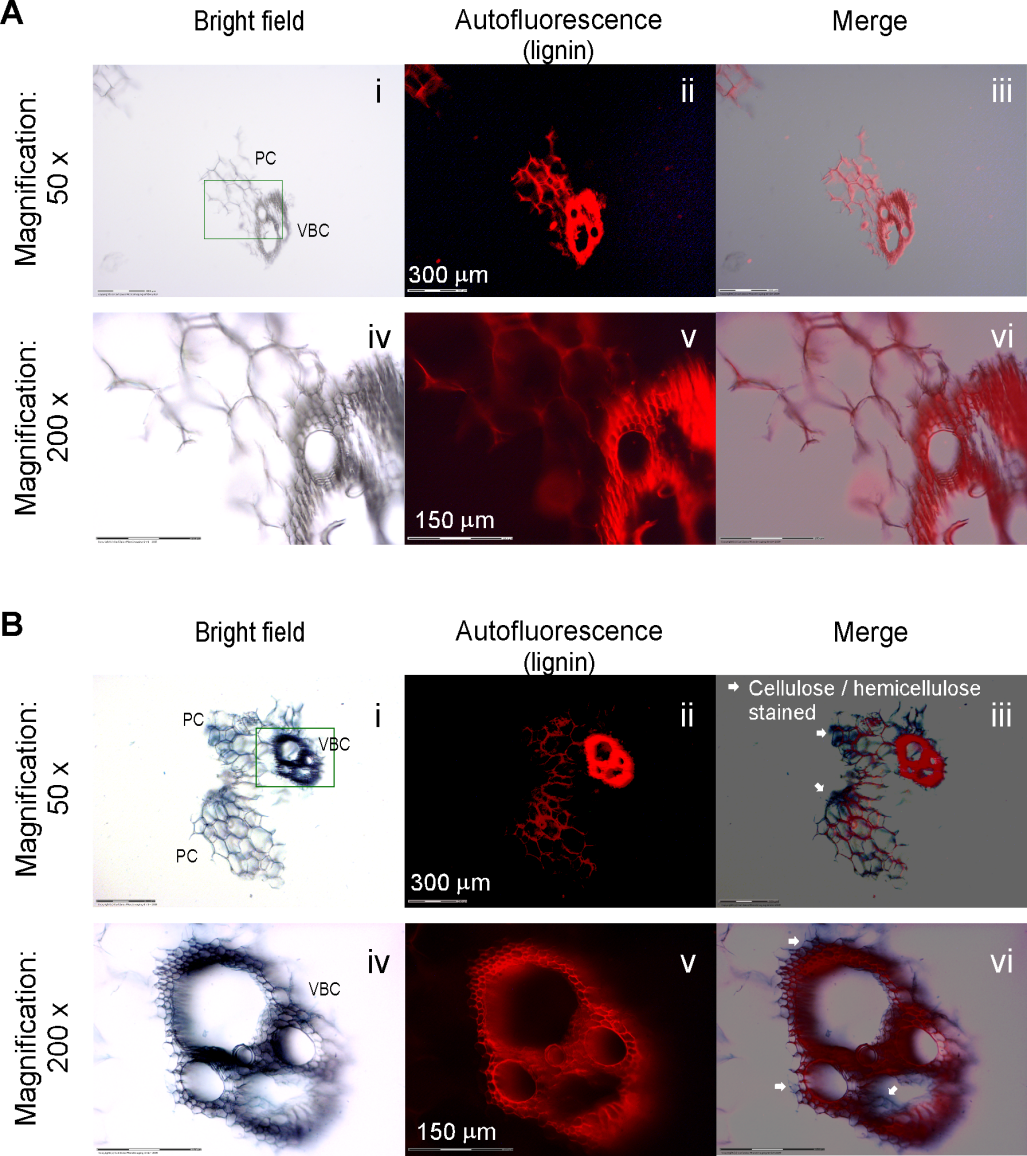
**Prussian blue staining of native, non-pre-treated corn stover with and without Fe^2+ ^incubation**. **(A) **Prussian blue staining of corn stover (CS) that went through incubation with 0.5 wt% H_2_SO_4 _but no Fe^2+ ^(negative control). **(B) **Prussian blue staining of corn stover that went through incubation with 0.5 wt% H_2_SO_4 _and Fe^2+^. Both incubations were conducted at room temperature. PC = parenchymal cells; VBC = vascular bundle cells.

The results are illustrated in Figure 6A, i and iv, which show the bright-field images obtained at ×50 and ×200 original magnification with Prussian blue staining of native, non-pre-treated CS samples (control). The red images represent the autofluorescence of lignin when the biomass was exposed to the UV laser light of the dissection microscope (Figure 6A, ii and 6v). The merged images were created by overlaying the red fluorescent image onto the image obtained following Prussian blue dye staining (Figure 6A, iii and vi). Overall, the control Prussian blue staining of CS without incubation with Fe^2+ ^ions verified that there is no nonspecific formation of blue precipitate in this Prussian blue staining of biomass procedure.

In contrast, Figure 6B, i and iv, show the bright-field images obtained with Prussian blue staining of native CS samples after incubation with 0.5 mM FeSO_4 _solution followed by washing. This may indicate the distribution of Fe^2+ ^ions following impregnation by soaking biomass in iron and/or acid prior to pretreatment. The Prussian blue staining is obvious and intense (compared with the control of no Fe^2+ ^incubation; Figure 6A, i and iv), showing the reaction with Fe^2+ ^ions present on the surface.

Furthermore, analysis of the other panels in Figure 6B reveals the differential deposition of Fe^2+ ^ions on the various components of plant cell walls in CS. First, a parallel examination of the Prussian blue staining in the bright field (Figure 6B, i and iv) and the red autofluorescence of lignin (Figure 6B, ii and v) shows that the dense staining of Prussian blue aligns with the location of lignin in the vascular bundle area, which is indicated by a rectangle in Figure 6B, i, and shown at high resolution in Figure 6B, iv. Second, the merging of the bright-field image and the red autofluorescence of lignin demonstrates that cellulose/xylan (located where there is very weak or no lignin red autofluorescence, as indicated by the white arrow in the right column of Figure 6B, iii and vi) also adsorbs Fe^2+ ^ions. Thus these results indicate that Fe^2+ ^ions associate with both cellulose/xylan and lignin in natural, untreated CS. Note that the above analysis is based on the assumption that where there is blue color but no red color, there is likely to be cellulose/xylan. Figure 6B shows the representative differential binding of Fe^2+ ^with lignin versus cellulose/xylan, and the images presented do not reflect the overall proportionate amount of cellulose/xylan in the CS biomass.

It is noteworthy that Prussian blue staining has been utilized for subcellular localization of bound iron ions in various organisms, including plants [15,16], but not to date in thermochemically pretreated biomass. Our current research is the first attempt to localize the metal ion in dilute acid/Fe^2+ ^ion-pretreated biomass.

### Imaging precipitation of Fe ions on the biomass surface following pretreatment

The pattern of Fe precipitation may provide clues to the role of iron salts in biomass pretreatment. Thus Prussian blue staining was conducted for detection of Fe ion deposition on pretreated CS. Because the pretreated biomass was not washed before being stained, the localization of Fe ions observed by Prussian blue staining is likely to reflect the *in situ *association of Fe with biomass, especially on the biomass surface.

**Figure 7:**
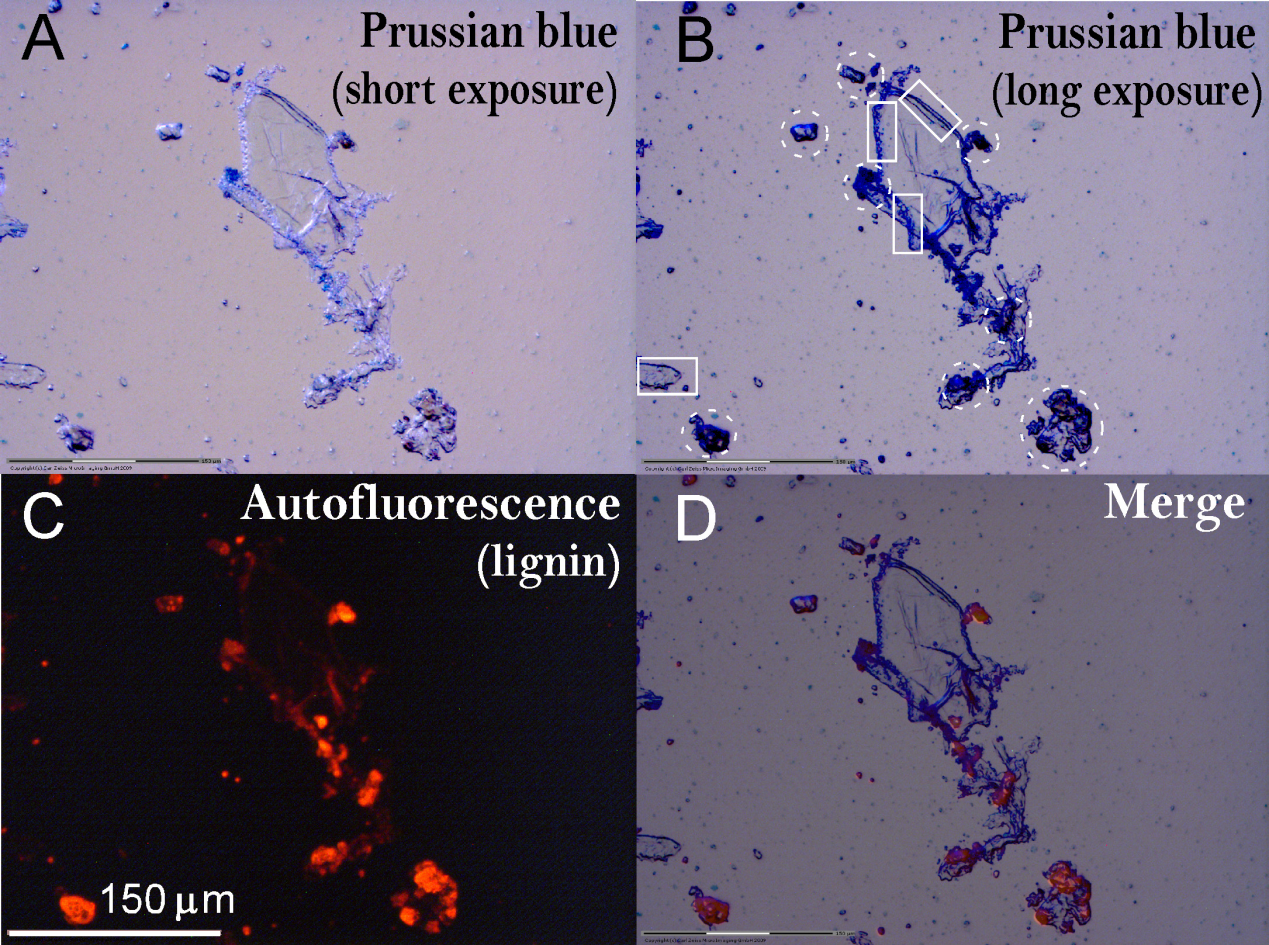
**Prussian blue staining of dilute acid/ferrous ion cocatalyst-pretreated corn stover**. **(A) **and **(B) **Bright-field images of Prussian blue staining of dilute acid/ferrous ion-pretreated corn stover (CS) using short and long exposure times with a microscope fitted with a camera, respectively. **(C) **Autofluorescence images of lignin in the same area shown above. **(D) **The overlay of Prussian blue staining and lignin autofluorescence images. For sample preparations, the milled CS was pretreated with 0.5 wt% sulfuric acid and 5 mM ferrous sulfate and pretreated at 150°C for 20 minutes. An approximately 100-mg aliquot was taken from well-mixed, pretreated samples and transferred to Prussian blue staining buffer using the regular Prussian blue staining and imaging procedures described in the Methods section.

The Prussian blue pigment formed was examined by laser dissection microscopy using a bright field, whereas lignin autofluorescence was observed at excitation wavelengths of 510 to 560 nm as red fluorescent light. On the basis of the comparison between the Prussian blue staining in the bright field (Figures 7A and 7B), the red autofluorescence of lignin (Figure 7C) and the merged image (Figure 7D), a large amount of iron ions seems to be associated with lignified regions of the biomass after dilute acid/Fe^2+ ^ion pretreatment, as illustrated by the dashed circles in Figure 7D. Furthermore, a substantial number of iron ions seem to be associated with regions of relatively scarce lignin content as indicated by the rectangle in Figure 7D (where there is weak or no red autofluorescence of lignin), likely the residual cellulose. These data are consistent with our observations in native, non-pre-treated CS (Figure 6), which indicate the association of Fe^2+ ^with lignin as well as cellulose/xylan.

### Transmission electron microscopy of dilute acid/Fe^2+ ^ion cocatalyst-pre-treated corn stover

**Figure 8:**
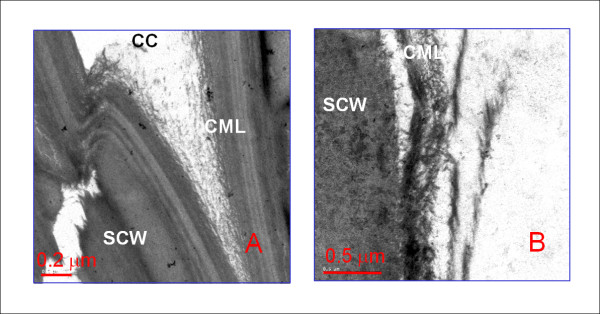
**Transmission electron microscopic photomicrographs of dilute acid/ferrous ion-pretreated corn stover pretreated at 150°C, 0.5 wt% H_2_SO_4 _+ 5 mM FeSO_4 _for 20 minutes**. **(A) **The cell corner regions including the exposed middle lamella and the primary cell wall display the most disruption of the lamellar cell wall structure. **(B) **Higher-magnification view of the delamination and fibrillation of the cell wall surface. CC = cell corner; CML = compound middle lamella; SCW = secondary cell wall.

TEM analysis of dilute acid/Fe^2+ ^ion cocatalyst-pretreated CS revealed unique patterns of cell wall delamination and surface erosion (Figure 8). The iron cocatalyst samples displayed a finely divided delamination pattern and some complete fibrillation of the cell walls, revealing isolated microfibrils. However, this physical disruption was limited to the compound middle lamellar region, which was especially evident at the cell corners and did not occur much beyond the primary wall into the bulk of the secondary cell wall (Figure 8).

### Scanning electron microscopy revealing surface ultrastructure of biomass

**Figure 9:**
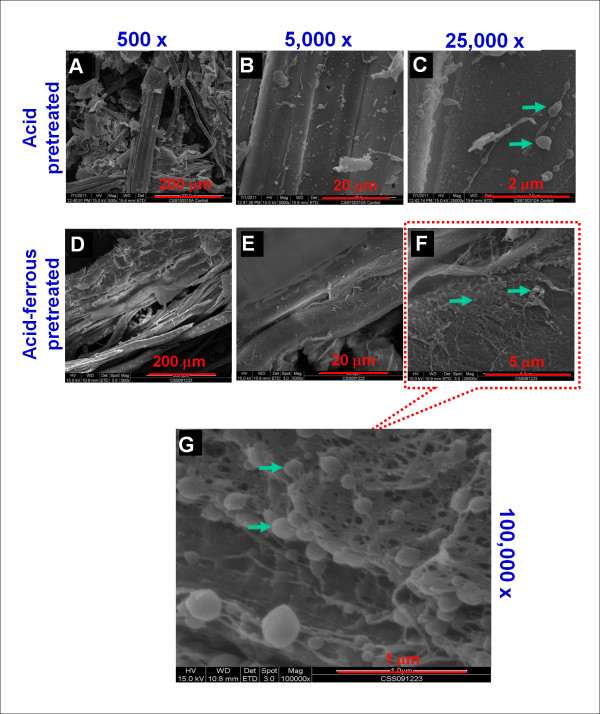
**Scanning electron microscopic images of dilute acid/Fe^2+ ^cocatalyst-pretreated corn stover**. **(A) **through **(C) **The top row shows images of dilute acid-pretreated corn stover (CS) controls from 150°C, 0.5 wt% H_2_SO_4_, 20-minute pretreatments. **(D) **through **(F) **The middle row shows images of CS samples from dilute acid/Fe^2+ ^cocatalyst pretreatment (that is, 0.5 wt% H_2_SO_4 _supplemented with 0.5 mM FeSO_4_) at 150°C for 20 minutes. **(G) **The bottom row shows a scanning electron microscopic image of dilute acid/Fe^2+ ^cocatalyst-pretreated corn stover at ×100, 000 original magnification. This image is an enlargement of a selected area of panel **(F)**. Arrows indicate lignin droplets.

In addition to TEM, we also performed SEM to reveal details of the surface features of the major components of biomass (that is, cellulose, xylan and lignin) after pretreatment. Figure 9 is a collage of SEM images obtained from samples of dilute acid-pre-treated CS and dilute acid/Fe^2+ ^cocatalyst-pretreated CS obtained from high solids (45 wt%) pretreatment in an NREL digester (National Renewable Energy Laboratory, Golden, CO, USA) at 150°C and 0.5 wt% H_2_SO_4 _for 20 minutes with versus without 0.5 mM Fe^2+^.

Overall, dilute acid-pre-treated and dilute acid/Fe^2+ ^ion-pretreated CS samples followed a similar structural modification in the vascular tissue, as shown in Figures 9A, B, D and 9E). However, dilute acid-pre-treated CS controls (top panels in Figures 9A through 9C) have smooth surfaces. The inside and outside surfaces of the pretreated samples appear smooth and littered with small to large droplets of lignin. In contrast, the surfaces of dilute acid/Fe^2+ ^ion-pretreated CS samples (lower panels in Figures 9D through 9F) appear to have a large percentage of the matrixing material (hemicelluloses) removed, with the cellulose macrofibers intact but floating above the surface in a delicate, lacelike pattern with interspersed lignin droplets (lower panels in Figures 9F and 9G). Lignin droplets are interspersed throughout the lacelike lattice as well as on the surface of the pretreated biomass sample (lower panels in Figures 9F and 9G).

In Figure 9G, a high-magnification image (×100, 000 original magnification) of a section of Figure 9F, the delicate, lacelike pattern of the putative cellulose macrofibers is readily apparent. Small lignin droplets are interspersed throughout the lacelike pattern. This removal of additional hemicellulose from the dilute acid/Fe^2+ ^cocatalyst-pre-treated CS may account for the enhancement in hemicellulose (xylose) yield following dilute acid/Fe^2+ ^cocatalyst pretreatment of CS compared to the control dilute acid alone pretreatment (Chen X, Shekiro J, Kuhn E, Wang W and Tucker MP, unpublished data). In addition, the lacelike fibrillation of the biomass surface following dilute acid/Fe^2+ ^cocatalyst pretreatment of CS may account for the enhancement in enzymatic digestion of the pretreated substrate compared with the digestion of dilute acid alone-pretreated CS controls (Chen X, Shekiro J, Kuhn E, Wang W and Tucker MP, unpublished data).

## Discussion

### Fe^2+ ^ions and cellulose component of biomass

In the present study, we investigated the effects and the role of Fe^2+ ^ions in the dilute acid pretreatment of FP and CL. As indicated by Raman spectroscopy, the Fe^2+ ^ions during pretreatment affect C-O bonds in polysaccharide backbone as well as the (-CH_2_OH) side groups of cellulose. These factors contribute to the depolymerization of cellulose and the reduction of its DP, eventually leading to the release of more simple sugars and the generation of more reducing sugar ends as well as making the biomass residues more digestible by enzymes.

### Fe^2+ ^ions and lignin component of biomass

Biochemical and imaging analyses revealed that, in addition to cellulose, Fe^2+ ^ions may interact with other plant cell wall components. Prussian blue staining was utilized to visualize the presence of and distribution of iron on the surface of the biomass, whereas SEM and TEM were carried out to reveal the structural details of the biomass after pretreatment. The observations made with Prussian blue staining suggest that Fe^2+ ^ions may remain in close proximity to heavily lignified regions of the biomass during pretreatment, and structural analysis by SEM and TEM revealed that these same regions are strongly affected morphologically by the presence of the Fe cocatalyst.

The formation and migration of spherical lignin deposits on the outer surfaces of fibers during pretreatment of CS have been described previously [17,18]. Our observation of smaller and more dispersed lignin droplets after dilute acid/Fe^2+ ^ion pretreatment suggests a possible role of iron in affecting the pattern of lignin droplet formation and migration during pretreatment. Further quantitative study is needed to confirm whether the production of smaller lignin droplets is another key contributing factor (in addition to the removal of xylan) that leads to better exposure of the structural carbohydrates that remain in the residual solid material after dilute acid/Fe^2+ ^iron pretreatment, facilitating the subsequent enzymatic digestion.

### Potential application of Fe^2+ ^ions in biomass pretreatment

First, although the results of this study are derived mainly from FP, CL and CS (which is derived from corn and/or maize, a monocot and a member of the grass family), we believe that the results we present are also applicable to woody biomass, such as poplar biomass. In fact, the initial report of Fe^2+ ^ion enhancement of biomass pretreatment was based on a woody plant biomass, the Douglas fir [1]. Second, in addition to the Fe^2+ ^ions, other transitional metal ions, such as Mn^2+ ^and Cu^+^, may have similar effects by enhancing biomass conversion during dilute acid pretreatment and are worth exploring. Third, in this study, we used gold-coated stainless steel reactors for the pretreatment of model cellulose materials and CS. This may have increased the cost of equipment required for using this technology at an industrial scale. Alternatively, a HASTELLOY corrosion-resistant alloy reactor (Haynes International, Inc, Kokomo IN, USA) may be an attractive option because it is resistant to sulfuric acid. Although a HASTELLOY C corrosion-resistant alloy reactor is 50% more expensive than a stainless steel SS316 reactor vessel [19], when the cost of the gold coating and, likely, recoating for stainless steel reactors are taken into consideration, a HASTELLOY corrosion-resistant alloy reactor seems more economical than gold-coated stainless steel in the long run.

To prevent the Fe^2+ ^ions from being oxidized to Fe^3+ ^ions prior to the FP, CL and biomass pretreatments, argon gas was used to purge the dilute acid/Fe^2+ ^solution. This argon-purging step may be a hurdle to clear for application at an industrial scale. The use of other forms of metal ions, such as Fe^3+^, that remain stable with exposure to air (oxygen) have been studied previously and may be an alternative worthy of further exploration.

## Conclusion

We employed a range of biochemical, spectroscopic and imaging techniques to investigate the impact of dilute acid/Fe^2+ ^ion pretreatment on the resultant digestibility of model cellulose substrates. By using a Prussian blue staining technique, we successfully observed the pattern of Fe^2+ ^binding with the cell wall components of native, non-pre-treated CS as well as the iron distribution after dilute acid/Fe^2+ ^ion pretreatment. Our imaging and FT Raman data suggest that the presence of ferrous ions during sulfuric acid pretreatment may affect multiple components of the plant cell wall, including the C-O-C and C-H bonds in cellulose. TEM and SEM imaging data suggest that ferrous ions, during pretreatment of CS, may improve xylan removal and lignin relocation. The comprehensive analyses presented in this article expand our understanding of the effects of dilute acid/Fe^2+ ^ion pretreatment; however, further investigation is needed to elucidate the detailed chemical mechanisms of this process, which will enable the optimization of this pretreatment strategy.

## Methods

An overview of the experimental approach we used, designed to investigate the metal ion-biomass interaction and effects on biomass conversion, is illustrated schematically in Figure 1.

### Model cellulose and biomass feedstock materials

Three types of model celluloses were used in current study. First, FP disks (150 mm in diameter, Whatman grade 1; Whatman Inc, Piscataway, NJ, USA) were cut into 1-cm × 6-cm strips and further trimmed to a final weight of 50.0 ± 0.1 mg for each strip. Second, acid-free and 99.5% lignin-free CL sheets (catalog no. 216; Arnold Grummer's Paper Making, Milwaukee, WI, USA) were cut into small disks using a 9-mm cork borer. The thick 2-mm disks were teased apart into thin disks, approximately 0.1 mm thick, to allow rapid and even diffusion of acid and cocatalyst throughout the CL disks. Third, Solka-Floc powdered cellulose 200 FCC was especially suitable for the DP studies because of its uniform, fine powder nature, which permitted reproducibility of DP measurements (described in the section of "Effect of Fe^2+ ^ions on cellulose degree of polymerization in acid pretreatment"). The CS used as our model biomass feedstock (variety 33A14; Pioneer Hi-Bred International, Inc, Johnston, IA, USA) was harvested at the Kramer Farm in Wray, Colorado. The whole CS was tub-ground at the site and further milled at NREL through a Mitts & Merrill rotary knife mill (model 10 × 12; Mitts & Merrill, Harvard, IL, USA) to pass through a ¼-inch screen.

### Pretreatments of model cellulose materials using pipe reactors

One-inch Swagelok 316L stainless steel tubing unions with two caps (Swagelok Co, Solon, OH, USA) were utilized as 20-ml pipe reactors. The inside surface of each reactor was plated with a very inert 99.97% gold coating at approximately 1-μm thickness to resist corrosion. It has previously been reported in the literature that chromium, molybdenum and a wide range of metal ions catalyze the destruction of sugars in solution [20]. The inert gold surface was used to prevent destructive catalysis by these metal ions and to ensure that any enhancements observed in pretreatment were the sole result of the cocatalyst incorporation during dilute acid pretreatments. The total volume of each pipe reactor was 20 ml, with minimal headspace remaining when the caps were tightened in place. For dilute acid/Fe^2+ ^ion cocatalyst pretreatment, the 0.5 wt% H_2_SO_4 _was prepared beforehand, then the acid solution was sparged with argon prior to adding the FeSO_4 _cocatalyst. The dilute sulfuric acid/Fe^2+ ^cocatalyst solution was added to the 250 mg of FP, CL or Solka-Floc powdered cellulose. We sparged the plated reactors with argon prior to adding the acid solution and sealing the reactors. The pipe reactors were immersed in a fluidized sand bath at the desired reaction temperature, and the residence time at the desired reaction temperature for the pretreatment was started when a separate control reactor equipped with a thermocouple came within 5°C of the desired reaction temperature.

After the pretreatments, no corrosion of the gold surface was found upon inspection of the reactors. The digestion mixture was poured out into preweighed 50-ml BD Falcon tubes (BD Biosciences, Franklin Lakes, NJ, USA). The tubes were spun in a centrifuge (model T-J-6; Beckman Coulter, Inc, Brea, CA, USA) at 3, 000 rpm for ten minutes. The supernatant was then transferred to a fresh tube, and the volume was measured. The supernatant (hydrolysate liquor) was used in the HPLC analysis to measure the released sugars, and the remaining FP and CL pellets were used in the DNS assay to measure the reducing sugar ends. For saccharification analysis, the remaining FP and CL pellets were washed three times with ddH_2_O and centrifuged between the washes. To observe the precipitation pattern of the remaining FP, ddH_2_O was added to the tubes containing the biomass residues. The tubes were then vortexed and left still overnight.

### Acid impregnation of corn stover

For control experiments, acid impregnation was carried out using 120 L of warm (40°C to 50°C) 0.5 wt% H_2_SO_4 _contained in a 200-L recirculation tank. For dilute acid/Fe^2+ ^ion cocatalyst impregnation, the acid was equilibrated for four hours in the recirculating bath, after which it was sparged with argon for 20 minutes and used to dissolve a measured amount of FeSO_4_. A HASTELLOY C-276 wire mesh basket (20-mesh screen; (Haynes International, Inc) was loaded with 14.5 kg of ¼-inch milled CS feedstock (approximately 94% solids) and lowered into the bath of warm dilute acid/Fe^2+ ^ion cocatalyst for two hours. A seal around the top of the basket forced acid to recirculate through the bed of CS. Following acid impregnation, the feedstock in the basket was drained of excess acid to approximately 20% solids and loaded into the mold of a hydraulic dewatering press (also sparged with argon), where the acid-impregnated feedstock was pressed to about 45% solids. The dilute acid/Fe^2+ ^ion cocatalyst-impregnated feedstock was prepared the same day as the pretreatment experiments were conducted to decrease the likelihood of oxidation of Fe^2+ ^to Fe^3+^, and the impregnated feedstock was kept under argon.

### Bench-scale pretreatment of corn stover

A 4-L steam explosion reactor made from HASTELLOY corrosion-resistant alloy was used for larger-scale high solids (approximately 45 wt% solids) pretreatment experiments. The reactor was prewarmed to pretreatment temperature and then sparged with argon for one minute using a lance that reached to the bottom of the hot reactor. Following sparging, the steam explosion reactor was loaded with 500.0 g of dilute acid/Fe^2+ ^ion cocatalyst-impregnated and pressed feedstock (about 45% solids) and quickly heated (approximately five to ten seconds) by direct steam injection to the reaction temperature. At the desired reaction time, the pretreated feedstock was rapidly blown into a flash tank and rapidly depressurized to atmospheric pressure.

### HPLC analysis for the sugars in hydrolysate liquors of pretreated filter paper and cotton linter

The pretreatment hydrolysate liquors were filtered through a 0.45-μm syringe filter prior to HPLC analysis for soluble carbohydrates. The filtrates were analyzed using a SP0810 column (Showa Denko America, Inc, New York, NY, USA) with a Micro-Guard De-Ashing column (Bio-Rad Laboratories, Hercules, CA, USA) at a flow rate of 0.6 ml/minute and a temperature of 80°C. The standards were used to identify the unknown HPLC peak areas by their retention time and to determine the sugar concentrations.

### Dinitrosalicylic acid assay for reducing sugar ends in filter paper and cotton linter residues

The total reducing sugars in pretreated cellulose residues were measured by DNS assay [21]. The assay was conducted on the scale of 0.2 g (dry weight basis) of washed sample of pretreated FP strips and CL disks. The washing step was performed to remove the iron ions or derived iron compounds from the FP and CL residues and to minimize any potential interference with the DNS assay. Because a glucose standard curve was used to calculate the sugar concentrations, the reducing sugars are expressed as "equivalent glucose."

### Enzymatic saccharification of the filter paper and cotton linter residues

The FP and CL residues from the pretreatment experiments first went through the extensive washing procedure critical for the determination of fraction insoluble solids [22]. Enzymatic digestions of washed, pretreated residues were then performed in 50 ml of 50 mM citrate buffer, pH 4.8, containing 1% substrate and 20 mg of GC 220 cellulase protein (Genencor, Rochester, NY, USA) per gram of substrate. The enzymatic reaction mixture was put into 125-ml Erlenmeyer shake flasks and incubated at 50°C and 130 rpm according to NREL LAP-009 [23]. No additional β-glucosidase was added. Samples of the slurries were taken at 1, 24, 48, 72, 96 and 168 hours. The glucose released was measured by HPLC.

### Determination of degree of polymerization in pretreated Solka-Floc cellulose

The molecular weight distributions of Solka-Floc cellulose samples were determined by SEC using a procedure modified from methods previously reported in the literature [24-26]. To permit dissolution in the SEC eluent tetrahydrofuran, cellulose samples were first carbanilated. Ten milligrams of a vacuum-dried cellulose sample were placed into a 5-ml reaction vial, and 2 ml of dry pyridine and 0.4 ml of phenylisocyanate were added. The reaction vial was kept at 70°C for 24 hours to complete the reaction. Methanol (0.4 ml) was added to the reaction mixture to react with the excess phenylisocyanate at the end of the reaction. The carbanilated cellulose was then precipitated in 26 ml of methanol/water (7:3 vol/vol), and the precipitate was washed twice with methanol/water (26 ml) before being dissolved in tetrahydrofuran (20 ml).

SEC was performed using five columns, 10^3^, 10^4^, 10^5^, 10^6 ^and 10^7 ^Å (PLgel columns; Agilent Technologies, Santa Clara, CA, USA), to cover the broad range of molecular weights of the cellulose samples. A calibration curve was obtained that allowed the conversion of retention times into estimated molecular weights by running polystyrene standards of known average molecular weight on the SEC system. Consequently, all molecular weights determined in this work are not absolute, but are relative to the calibration curve. The conditions for chromatography were as follows: flow rate 1.0 ml/minute, UV detector wavelength 235 nm (with a bandwidth of 10 nm), injection volume 50 μl and column temperature 25°C. Cellulose DP was calculated by dividing the apparent molecular weights by 519 (molecular weight of a repeating unit of carbanilated cellulose with the degree of substitution of 3.0).

### Fourier transform Raman spectroscopy and challenge in analyzing pretreated biomass

Lignin, which constitutes a significant portion of dilute acid-pre-treated CS (typically containing 27% to 31% lignin in dry weight) [27], poses a challenge while conducting FT Raman analysis on these samples. The lignin-caused dark coloration of pretreated CS may lead to the burning of biomass at high power levels. Alternatively, it may not generate enough signal at safe levels. In addition, our trial experiments in conducting FT Raman analysis of pretreated CS showed that the Raman spectra vary with different thickness of sample (which affects signal magnitude) and also for different areas of sample, which causes peak shifting (data not shown). For these reasons, we decided to use FP and CL as model celluloses instead of using biomass, such as CS, to conduct FT Raman analysis.

FT Raman data were collected using a Nicolet Raman 960 spectrometer (Nicolet Instrument Corp/Thermo Fisher Scientific, Madison, WI, USA) with 1, 064 nm of Nd:YVO_4 _laser excitation and a Ge detector. Samples were pressed onto glass coverslips and mounted on an upright holder. Measurement parameters included 256 scans at 8 cm^-1 ^resolution with approximately 200-mW excitation power.

### Prussian blue staining

The principle of Prussian blue staining is that in the presence of hydrochloric acid, iron ions will form a "Prussian blue" precipitate of ferric ferrocyanide in reaction with potassium ferrocyanide, the component of Prussian blue staining buffer. This blue precipitate will be deposited, and can be observed, in the cell wall sites where the iron ions are located.

We used Prussian blue staining first to detect the presence of Fe^2+ ^ions in native, non-pre-treated CS and second to examine the deposition of Fe^2+ ^ions on the different components of plant cell walls in dilute acid alone- versus dilute acid/Fe^2+ ^ion-pretreated CS. For the first purpose, dry, native, non-pre-treated CS samples were wetted and hand-sectioned, then quickly transferred to either 0.5 wt% H_2_SO_4 _(for control) or 0.5 wt% H_2_SO_4 _+ 1 mM Fe_2_SO_4 _and incubated in a shaker at room temperature for two hours before being subjected to the Prussian blue staining procedure.

For the second purpose, the dilute acid alone- versus dilute acid/Fe^2+ ^ion-pretreated CS samples were directly hand-sectioned and then subjected to the following Prussian blue staining procedure. The hand-cut CS materials were washed twice in 200 mM NaCl and suspended in Prussian blue staining buffer (2% potassium ferrocyanide, 2% hydrochloric acid) for 45 minutes in the dark at room temperature, followed by one or two washes with distilled water. The stained materials were mounted on glass slides and imaged using a PALM laser dissection microscope (Carl Zeiss MicroImaging GmbH, Jena, Germany). Adobe Photoshop software (Adobe Systems, Inc, San Jose, CA) was used to overlay the Prussian blue-stained images with the autofluorescence images of lignin.

### Laser dissection microscope

The PALM laser dissection inverted microscope was used for bright-field and fluorescence imaging. This system consists of a UV laser and an inverted microscope equipped with fluorescence optics, including filter sets for work with green, red or other fluorescent markers. The original magnification of the images was ×50, ×200 and ×400. The Prussian blue-stained dilute acid (control) and dilute acid/Fe^2+ ^ion-pretreated CS biomass samples were placed in slides on the stage and illuminated using the bright-field and fluorescence optics. The fluorescence images were obtained where the samples were illuminated using the UV laser.

### Scanning electron microscopic imaging

SEM imaging was performed using a Quanta™ 400 FEG high-resolution low-vacuum scanning electron microscope (FEI Co, Hillsboro, OR, USA) under low-vacuum (0.40 to 0.65 Torr) operating conditions with a gaseous solid-state detector. For SEM observations, the pretreated CS samples were prepared for imaging by freezing them in liquid nitrogen and then freeze-drying. This freeze-drying step was performed to prevent potential structural distortion that can be caused by the surface tension of liquid water during regular evaporation at atmospheric conditions. The samples were then mounted on aluminum stubs for SEM examination. Imaging was performed at beam-accelerating voltages ranging from 12.5 to 25 keV. Images were obtained at original magnifications ranging from ×500 to ×100, 000.

### Transmission electron microscopy imaging

Samples were fixed twice for six minutes each with variable power in 2.5% glutaraldehyde buffered in 0.1 M PBS (Electron Microscopy Sciences, Hatfield, PA, USA) under vacuum pressure. The samples were dehydrated by treating them with increasing concentrations of ethanol for one minute at each dilution (30%, 60%, 90% and 3 × 100% ethanol). After dehydration, the samples were infiltrated using the EMbed 812 Resin for Microscopy kit (catalog no. 14120; Electron Microscopy Sciences) for three minutes under vacuum pressure with one step at room temperature overnight in increasing concentrations of resin (15%, 30%, 60%, 90% and 3 × 100% resin, diluted in ethanol). Infiltrated samples were transferred to flat-bottomed TAAB capsules (TAAB Laboratories Equipment Ltd, Aldermaston, UK) and polymerized in a nitrogen-purged vacuum oven at 70°C for 24 hours.

We sectioned embedded samples to approximately 60 nm by using a diamond knife (DiATOME US, Hatfield, PA, USA) on an Ultramicrotome Leica EM UC7 (Leica Microsystems, Wetzlar, Germany). Sections were collected on 0.5% Formvar-coated copper slot grids (SPI Supplies, West Chester, PA, USA). All grids were poststained for six minutes with 2% aqueous uranyl acetate and for three minutes with 1% KMnO_4 _for ten minutes to selectively stain for lignins. Images were taken with a 4-megapixel Gatan UltraScan 1000 camera (Gatan, Inc, Pleasanton, CA, USA) mounted on a Tecnai™ G^2 ^20 Twin 200-kV LaB_6 _Transmission Electron Microscope (FEI Co).

## Abbreviations

CL: cotton linter; CS: corn stover; ddH_2_O: distilled deionized water; DNS: dinitrosalicylic acid assay; DP: degree of polymerization; FT Raman spectroscopy: Fourier transform Raman spectroscopy; FP: filter paper; HPLC: high-performance liquid chromatography; PBS: phosphate-buffered saline; SEC: size exclusion chromatography; SEM: scanning electron microscopy; TEM: transmission electron microscopy.

## Competing interests

The authors declare that they have no competing interests.

## Authors' contributions

MPT and MEH designed and coordinated the study and revised the manuscript. HW and MPT conducted the pretreatments, biochemical assays and Prussian blue staining of samples and prepared the manuscript draft. BSD, PNC and TBV carried out the TEM and SEM experiments. WW and DKJ studied the effects on particle size and degree of polymerization in pretreated samples. LMG obtained the FT Raman spectroscopy spectra of samples. SYD, BSD, PNC and YZ contributed to data analyses and manuscript preparation. All authors read and approved the final manuscript.
